# Dietary Diversity, Household Food Insecurity and Stunting among Children Aged 12 to 59 Months in N’Djamena—Chad

**DOI:** 10.3390/nu15030573

**Published:** 2023-01-21

**Authors:** Goudja Gassara, Qian Lin, Jing Deng, Yaxi Zhang, Jieqiong Wei, Jihua Chen

**Affiliations:** 1 Department of Nutrition Science and Food Hygiene, Xiangya School of Public Health, Central South University, Changsha 410008, China; 2 Department of Epidemiology and Health Statistics, Xiangya School of Public Health, Central South University, Changsha 410078, China; 3 Hunan Provincial Key Laboratory of Clinical Epidemiology, Changsha 410078, China

**Keywords:** children dietary diversity, household food security, stunting, Chad

## Abstract

Background: Household food insecurity is increasingly recognized as a global health problem, particularly in sub-Saharan Africa. This study aimed to contextualize the associations between household food insecurity, dietary diversity and stunting in N’Djamena. Methods: This study is a community-based cross-sectional study, and the SMART (Standardized Monitoring and Assessment of Relief and Transitions) methodology was used to calculate the sample size. A total of 881 households were selected for the survey. A 24-h recall evaluated the dietary diversity score (DDS), the Household Food Insecurity Access Scale (HFIAS) made it possible to assess household food insecurity (HFI), and stunting among children aged 12 to 59 months was assessed by anthropometric measurements. Logistic regression was constructed to determine the association between household food insecurity, dietary diversity, and stunting. The study was conducted from January to March 2022. Results: The prevalence of severe food insecurity was 16.6%, and that of stunting was 25.3%. The mean DDS was 6.5 ± 1.6. Severe food insecurity (OR 2.505, CI: 1.670–3.756) was significantly associated with stunting. The association between DDS and stunting was not significant. Conclusions: This study’s prevalence of household food insecurity and stunting was very high. Household food insecurity and household size were significantly associated with stunting.

## 1. Introduction

Household food insecurity is increasingly recognized as a global health problem, particularly in sub-Saharan Africa [1]. It is one of the major underlying causes of child malnutrition [2]. It is defined as “an economic and social condition at the household level of limited or uncertain access to adequate food” [3]. The Household Dietary Diversity Score (HDDS) measures the total number of food groups consumed in the past 24 h by a household member, including foods prepared at home but eaten away, such as a lunch bag [4,5].

Like many countries in the Sahelian zone, Chad experiences unfavorable climatic conditions to which equally precarious socio-economic factors can be added. There are few studies on household food insecurity in African cities. However, some research has been able to describe this food insecurity because it is very evident in African cities. In South Africa, for example, a study showed that 80% of households were forced to limit the variety and amount of food consumed [6], and a study conducted in Ethiopia, specifically in Addis Ababa, showed that average food insecurity and severely affected one in two people in a population of social workers [7].

Poor infant and young child feeding (IYCF) practices have been identified as critical proximate causes of stunting, and these practices include a lack of exclusive breastfeeding to 6 months of age and limited dietary diversity throughout infancy and early childhood [8,9,10].

In 2018, an assessment of household food security was carried out in the city of N’djamena by the Chadian Ministry of Agriculture and the Environment, and it emerged from this survey that 24% of households were food insecure, of which 2% were in severe food insecurity [11]. According to the national nutrition survey, the national prevalence of stunting was 32.4% in 2017, 31.9% in 2018, 32% in 2019, 30.5% in 2020 and 30.4% in 2021 [12]. The prevalence of the percentages shows an improvement in nutrition, but this nutritional situation is still alarming according to the World Health Organization (WHO) classification.

Several studies have confirmed the association between stunting and household food insecurity [13,14,15,16]. However, few studies have assessed household food insecurity, child dietary diversity, and stunting in N’djamena. The lack of study on the relationship between household food insecurity and dietary diversity with stunting in N’djamena prompted us to initiate this study to contextualize the associations between household food insecurity, dietary diversity, and stunting. This study was carried out from January to March. Household food insecurity (HFI), measured by the Household Food Insecurity Access Scale (HFIAS), was included in this study as a variable. A 24 h recall was also used to assess child dietary diversity among children aged 12 to 59 months, and anthropometric measurements to assess stunting among children.

## 2. Materials and Methods

### 2.1. Study Design

It is a cross-sectional study using the SMART methodology (Standardized Monitoring and Assessment of Relief and Transitions) [17]. During the survey, we used a 2-stage cluster sampling design based on probability proportional to population size and thus obtained a representative sample of our study population. A standardized methodology for undertaking surveys, SMART collects information on two vital public health indicators: the nutritional status of children under five and the mortality rate of the population. These 2 indicators make it possible to assess the severity of a humanitarian crisis.

### 2.2. Study Setting

This study was conducted in the city of N’Djamena (the capital of the Republic of Chad). It is located in the center-west of Chad. N’Djamena is subdivided into ten (10) administrative units called “Arrondissements” and includes 4 health districts [18]. Its area is estimated at 104 km². The total population of N’djamena was estimated in 2021 at 1,676,257 inhabitants (259,964 children aged 12–59 months) distributed in 335,251 households [18].

About 80% of N’Djamena’s population works in agriculture-based industries, including crop and livestock [19,20]. N’Djamena has a hot semi-arid climate with a long dry season and a short rainy season from June to September. The study was conducted between January and March 2022.

### 2.3. Study Population

All children aged 12 to 59 months residing in the study area during the last 6 months were included. However, any child under 12 or over 59 months with a severe medical problem, a physical malformation, or not living in the study area was excluded from the sample.

### 2.4. Sample Size and Sampling Techniques

ENA (Emergency Nutrition Assessment) software for SMART (9 July 2015 version) was used to calculate the sample size [21]. The sample was determined on the upper bound of the prevalence of the SMART survey in Chad carried out in September 2021, the desired precision of 5% and a design effect of 2, an average household size of 5, a percentage of children under five in the population (15%) and non-response rate estimated at 10% [12]. Thus, 900 households were selected for the survey thanks to 60 clusters, and 900 children aged 12 to 59 months were included in the study.

### 2.5. Data Collection and Variable Measurement

We used pre-designed and pre-tested survey forms to interview the participants in the study. We thus obtained information on the socio-demographic characteristics of the household, head of the family, and the children (age and sex of household head, age and sex of the children, religion, marital status, household size, level of education, profession of household head, and socio-economic status of the household). We also obtained information on household food insecurity, dietary diversity, and stunting.

Participants were interviewed using the standardized survey form. Interviewers and supervisors were rigorously trained for two (2) days by the research team. Before data collection, interviewers and supervisors conducted a preliminary survey in a community other than those targeted for the survey. The pre-survey was conducted with 100 randomly selected households. Participant feedback before the survey was collected, and the questionnaire was improved and revised. Supervisors reviewed each survey sheet for completeness and consistency at the end of each survey day. Regular adjustments have been made, particularly concerning anthropometric measurements.

The construction of the socio-economic status (SES) of the household will be based on some variables, including sources of electricity for lighting and cooking, electrical equipment (television, radio, refrigerator, mobile phone, computer), sources of water and heat used in households (tap water, borehole water), types of dwelling (red brick house, cinder block house) and latrines. The index will be divided into three classes to have the different economic levels of the household (low SES, middle SES, and high SES) [22,23].

HFI was assessed by the Household Food Insecurity Access Scale (HFIAS), developed by the United States Agency for International Development [24]. HFI is thus classified into four degrees: food security, mild food insecurity, moderate food insecurity, and severe food insecurity. Food security applies if the households had experienced no insecure food conditions or had rarely worried about not having enough food.

The method of collecting dietary diversity information described here consists of a 24-h recall of all foods and beverages consumed by children aged 12–59 months. The procedure recommended by the Food and Agriculture Organization (FAO) of the United Nations was used to assess dietary diversity [25]. Twelve food groups are proposed for the Dietary Diversity Score (DDS). DDS are calculated by counting the number of food groups consumed by the child in the household during 24 h. The value of this variable is between 0 and 12, i.e., 1 point for each food consumed, 2 points for two foods consumed, and so on, with a maximum score of 12 points. The score of the 12 food groups is obtained by adding the value of the variables reflecting the 12 food groups. For each child, the variable can take any value between 0 and 12. A child would score 0 if he ate none of the 12 food groups and 12 if he received foods from 12 food groups. In this study, based on the median 5 of the DDS, a score of DDS > 5 was judged as a good level of dietary diversity and a score of DDS ≤ 5 as a low level of dietary diversity.

The anthropometric status of children will be determined using Z-scores from the World Health Organization (WHO) Growth Standards [26]. All children were measured for height and weight. Thus, for weight gain, the children were weighed on a digital electronic scale and recorded in kilograms to the nearest 0.1 kg, with light clothing and no shoes [27]. We used a portable stadiometer to measure length and height. For children over 2 years old, height was measured in a lying position and a standing position for children over 2 years old. Stunting is a height-for-age (HAZ) Z-score <–2 SD of the median WHO Child Growth Standards.

### 2.6. Statistical Analysis

Anthropometric data were analyzed using ENA for SMART to obtain the prevalence of stunting based on Z-scores according to the World Health Organization (2006) Growth Standards Z-scores. Then, for an in-depth study, the data were exported to IBM SPSS Statistics version 22.0 software (IBM Corp., Armonk, NY, USA). The results of the analyses concerning the quantitative variables will be presented in the form of a number, average, standard deviation, minimum and maximum, median, and a graphical representation if relevant.

A bivariate and multivariate logistic regression analysis investigated factors associated with stunting.

Statistically significant variables in bivariate analysis were included in the multivariate analysis. The bivariate and multivariate logistic analysis results were presented using crude and adjusted odds ratios (odds ratio, OR) and 95% confidence intervals (CI). The level of significance of the associations retained was 5%.

### 2.7. Ethical Consideration

The study was conducted according to the guidelines of the Declaration of Helsinki and approved by the ethics approval committee of the Xiangya School of Public Health, Central South University (reference number XYGW-2021-111, 12/28/2021). Informed consent was obtained from all participants involved in the study. Informed consent was obtained from all subjects involved in the study, and they were informed of their obligation to discontinue or refuse to participate in the study.

## 3. Results

Table 1 shows the characteristics of household heads. From a total of 900 households surveyed, 881 households (i.e., a response rate of 97.9%) gave complete responses. The majority (98.1%, *n* = 864) of household heads were married, and more than two-thirds (70.1%, *n* = 618) of them were men (Table 1). Nearly two-thirds (61.9%, *n* = 545) of household heads were under 35 years old, with an average age of 34.35 ± 7.15 years. About 68% of respondents reported having an education level above the secondary level, and 32% of household heads had completed primary school. About 47% of household heads were wage-earner (public or private sector), 11% were unemployed, and 42% were self-employed.

Table 2 shows the characteristics of households. Two out of three households (62%, *n* = 543) comprised of six to eight people. Nearly sixty-eight percent of households supplied drinking water from borehole water, 29.4% from municipal taps, 1.9% from wells, and 0.2% consumed surface water. Most households had one or two children aged 5 years or less. Economically, almost half (53.3%) of households had a single source of income and lived in houses built in red bricks (55.3%). About 11% and 87% of households had low and middle socio-economic status, respectively.

Table 3 shows the characteristics of children included in the study. In total, 40.5% of children under 5 were male compared to 59.9% of females. The 12–23-month age group had 547 children (62.1%), and the 24–59 month age group had 334 children (37.9%). The mean age was 22.51 ± 7.9 months. A total of 25.3% of children under 5 were stunted, and the portion of children aged 12–23 months was most at risk of stunting (27.8% versus 21.3%, *p* < 0.031) (Figure S1).

**Figure 1:**
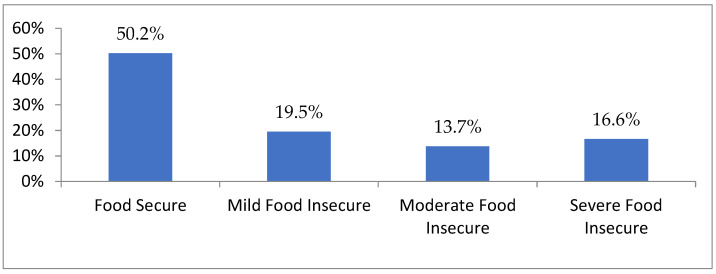
State of food security of households living in central-western Chad, January 2021.

Figure 1 shows the prevalence of household food insecurity in N’Djamena. This study found that of the total households inquired, 146 (16.6%) households were in severe food insecurity, and 442 (50.2%) households were food-secure. Food insecurity was higher in households led by men than in those led by women (18.9% against 11%, *p* < 0.001), and almost two-thirds (58.8%) of households led by unmarried couples were in severe food insecurity against those married (15.7%).

Table 4 shows the dietary diversity of children. Of the 881 children aged 12–59 months included in the study, 241 (27.4%) of the children had not reached the minimum score (minimum of five or more food groups in the last 24 h). In this study, the mean DDS is 6.5 ± 1.6. Of the 241 children who did not reach the minimum score, 173 (31.6%) were aged 12–23 months, and 68 (20.4%) were aged 24–59. Cereals (99.10%) were the food group most consumed by children, followed by sugars and sweets (98.1%) (Figure S2).

Table 5 below shows children with and without stunting characteristics. This study revealed that the prevalence of stunting among children living in an unmarried household was 47.1%, compared to 52.9% of households with children without stunting (*p* = 0.072). The prevalence of stunting was 42.6% among children living in households with at least eight members (*p* = 0.003). We found that the prevalence of stunting was 27.8% among children aged 12–23 months, compared to 21.3% among those aged 24–59 months (*p* = 0.031).

Of households with stunted children, 25.9% of household heads were men, 24.9% were married, 27.3% were employed, and 25.5% of households had only one (1) source of income. The chi-square test showed significant differences in stunting at marital status, household size, and child age.

Table 6 reports the logistic regression results for children aged 12–59 months with and without stunting. This study showed many associations between stunting and specific variables, but most of the associations were not significant.

Children living in a household with more than eight members were 2.913 times likely to be stunted (OR 2.913, CI: 1.528–5.552) than those living in a household with less than eight members.

In our study, stunting was significantly associated with children aged 12–23 months (AOR 1.428, CI: 1.033–1.973).

Children aged 12–59 months living in a mildly (AOR 1.609, CI: 0.987–2.621) and a severely food insecure household (OR 2.505, CI: 1.670–3.756) were more likely to be stunted than those living in a food-secure household.

There is no statistically significant association between children who did not achieve minimum dietary diversity, stunting, and children aged 12–59 months living in an unmarried household.

## 4. Discussion

Of the 881 children surveyed, 25.3% suffered from stunting, and this prevalence is qualified as high (20 to <30%) according to WHO standards. In 2020, according to the WHO, 149.2 million children under the age of five, or 22% of children, suffer from stunting worldwide [28]. This study’s stunting rate was lower than the 27.1% found in the Asia–Pacific region in 2021 [29]. This prevalence is also lower than that of studies carried out in Thailand, Pakistan, Burkina Faso, Rwanda, Tanzania, and Ethiopia, which found 38%, 50.7%, 32.48%, 37.6%, 52.8%, 37.5%, 32.8% and 38%, respectively [14,16,30,31,32,33,34,35]. However, our results were superior to those of other studies conducted in Vietnam, Mali, Benin, Senegal, and China, which found 25%, 14.1%, 24.2%, 16.6%, and 20.7%, respectively [13,36,37,38,39]. However, it is important to note that studies have noted breed differences in body weight and fatness [40,41,42].

Our study found that household food insecurity is high in N’Djamena. Thus, it was found that 16.6% of households were in severe food insecurity. Our results were superior to those of studies conducted in the United States, Ethiopia, and Kenya [39,43,44]. However, similar studies conducted in South Africa and Iran had found results inferior to ours [45,46]. This prevalence shows that food insecurity is a severe problem in N’Djamena. The results of an assessment of household food security in N’Djamena by the Chadian Ministry of Agriculture and the Environment showed that 50.8%, 22.0%, and 1.9% of households were mildly, moderately, and severely food insecure, respectively [11], and only 25.3% of households were food secure. Our survey data found that 19.5%, 13.7%, and 16.6% of households were mildly, moderately, and severely food insecure, with 50.2% in food security. Our results were superior regarding food security and severe household food insecurity. These results could be explained by the fact that our study used a larger sample size than the assessment mentioned earlier, and the methodology used for the household food security assessment, which is also different from ours.

The study also evaluated the relationship between stunting and HFI in children aged 12 to 59 months in N’Djamena. Our results show that there is a significant association between HFI and stunting. It was found that children from severe HFI households were more at risk of being stunted than those from food-secure households. This result was consistent with similar studies in Thailand, Ethiopia, Bangladesh, Malaysia, Brazil, Rwanda, and India [14,15,47,48,49,50,51]. Other similar studies found no association between these two factors [52,53,54]. This difference could be explained by the different groups of children included in the different studies. The sample used in our study was children aged 12–59 months, while some studies used age groups of 6–23 months.

In developing countries, dietary diversity increases energy and micronutrient intake [55]. Thus, dietary diversity is considered a good predictor of good dietary quality and micronutrient density in children [56,57]. This study observed high consumption of foods from the cereals and sugars/sweets group. Studies in Tanzania found grains and cereals to be infants’ first complementary foods [58,59,60,61]. Other research has observed, on the contrary, a high consumption of vegetables. For example, a Kenyan study found 100% vegetable consumption [62], and a South African study found that starchy foods were the most consumed [63].

On the other hand, meat, milk, and egg consumption were relatively low among children aged 12–59 months in N’Djamena. However, these foods of animal origin contain a variety of micronutrients (riboflavin, iron, calcium, zinc, vitamin A and vitamin B-12) that are difficult to obtain from foods of plant origin alone [64]. Therefore, insufficient intake of these foods could lead to stunting [65].

The average DDS of our study was 6.5 ± 1.6, thanks to the FAO notation of 12 food groups over a reference period of 24 h. The average DDS (6.5) found in our study was almost similar to other studies conducted in China using 9 food groups (5.77%) [66], in Nigeria using 12 food groups (6.04%) [67], and in South Africa (6.52%) using 9 food groups [68]. Other similar studies conducted with children under five had found lower average values, such as in South Africa (4.39) [63], Trinidad and Tobago (4.6) [69], Sri Lankan (4.56) [70], and Filipino (4.91) [55]. The ages of children in the studies, the types and groups of foods, and the differences in scoring systems often make comparisons between countries difficult, so it is essential to be cautious about interpreting the DDS.

In our survey, children’s DDS were not significantly associated with stunting. Our results are consistent with studies conducted in Ethiopia that did not find an association between DDS and HAZ scores [71,72]. However, a study in Kenya showed that higher DDS was associated with lower levels of stunting in children aged 24–59 months [73]. Other studies have also found this association between dietary diversity and stunting [53,74,75,76]. Some studies have confirmed that poor diet cannot be the only risk factor for malnutrition and draws attention to the fact that genetics can also directly influence malnutrition [77,78,79]. Stunting begins early in a child’s life and reflects longer-term nutritional status [80]. Therefore, we must insist on improving dietary diversity in children at an early stage of life, thus preventing the onset of stunting.

In this survey, children from households with at least eight members were twice as likely to be stunted. In Indonesia, a survey showed that a larger household size was associated with a lower likelihood of child stunting in urban areas. In contrast, in rural areas, it was associated with a lower likelihood of stunting [81]. Similar studies in Ethiopia and the Marshall Islands [82,83] also found a significant association between these two factors.

Our study also revealed a significant relationship between the child’s age and stunting. Children aged 12 to 23 months were the most affected by stunting, such as in Bangladesh and the Marshall Islands [83,84]. However, our data contrast with other studies from Tanzania, Kenya, Nigeria, and Nepal, where children aged 24–59 months were more likely to be stunted than younger children [33,85,86,87]. This result could be explained by the fact that the children were in the weaning period and, therefore, more exposed to diseases likely to create a nutritional imbalance.

This study has several limitations. A cross-sectional design, so evidence from this study does not show causality. Data regarding dietary diversity had been collected only for one recall (24-h recall). Some data from our study were excluded due to missing information or refusal, which could have introduced selection biases and affected the results’ representativeness in a broader context for Chad. The information provided by the population in a situation of insecurity and assuming that the declaration of food consumption gives a chance of any food or financial support could also introduce a potential bias. In addition, the study provides evidence of association with stunting that can be used to suggest recommendations. To our knowledge, this is the first study to assess the relationship between household food insecurity and dietary diversity with stunting among children aged 12–59 months in N’Djamena.

## 5. Conclusions

The study concludes that the prevalence of stunting in our research is classified as high according to WHO standards. This study shows that stunting children were not significantly associated with low dietary diversity and marital status. However, stunting in children aged 12–59 months was significantly associated with household food insecurity, child’s age, and household size.

Our results will thus make it possible to develop or design recommendations for implementing the multisectoral nutritional intervention, particularly at the community level, and create income-generating mechanisms to reduce malnutrition and household food insecurity in N’Djamena. The nutritional status of children under five must be continuously monitored for early detection and management of malnutrition. Further research should be considered to understand the causes and pathways leading to these associations with stunting.

## Figures and Tables

**Table 1:** Demographic characteristics of household heads (*n* = 881).

Characteristics	Modality	*n* (%)
Age of household head	≤35 36–45 46–55 ≥56	545 (61.9) 297 (33.7) 24 (2.7) 15 (1.7)
Sex of household head	Male Female	618 (70.1) 263 (29.9)
Marital status of household head	Married Otherwise	864 (98.1) 17 (1.9)
Education of household head	Primary level and below Secondary level and above	283 (32.1) 598 (67.9)
Profession	Wage-earner Self-employed Unemployed	414 (47.0) 370 (42.0) 97 (11.0)

**Table 2:** Demographic characteristics of households (*n* = 881).

Characteristics	Modality	*n* (%)
Household income source	≤1 2–4	470 (53.3) 411 (46.7)
Household size	≤5 6–8 >8	291 (33) 543 (61.6) 47 (5.3)
Number of Children ˂ 5 years	1–2 3–4 5–6	826 (93.8) 50 (5.7) 5 (0;5)
Household drinking water source	Tap water Well water Borehole water Surface water	259 (29.4) 17 (1.9) 603 (68.5) 2 (0.2)
Type of accommodation	Rammed earth house Red brick house Cinder block house	156 (17.7) 487 (55.3) 238 (27.0)
Household socio-economic status	Lowest Middle Highest	93 (10.6) 763 (86.6) 25 (2.8)

**Table 3:** Characteristics of children included in the study (*n* = 881).

Variable	*n* (%)	Mean ± SD
Child’s sex Boys Girls	357 (40.5) 524 (59.5)	
Child’s age 12–23 months 24–59 months	547 (62.1) 334 (37.9)	22.5 ± 7.9

**Table 4:** Distribution of children aged 12–59 months by dietary diversity score.

Variable	*n* (%)	Mean ± SD
DDS Minimum not met Minimum met	241 (27.4) 640 (72.6)	6.5 ± 1.6

**Table 5:** Associations of child stunting status with socio-demographic features of the respondents in central-western Chad, January 2021 (*n* = 881).

Characteristics	Stunted (*n* = 223)			No Stunted (*n* = 658)		*p*-Value
	n	%		n	%	
Sex of household head						
Male	160	25.9		458	74.1	0.545
Female	63	24.0		200	76.0	
Marital status of household head						
Married	215	24.9		649	75.1	
Otherwise	8	47.1		9	52.9	0.072
Level of education of household head						
Primary level and below	66	23.3		217	76.7	
Secondary level and above	157	26.3		441	73.7	0.350
Profession						
Wage-earner	113	27.3		301	72.7	
Self-employed	84	22.7		286	77.3	
Unemployed	26	26.8		71	73.2	0.315
Household income source						
≤1	120	25.5		350	74.5	
2–4	103	25.1		308	74.9	0.873
Household size						
<5	59	20.3		232	79.7	
5–8	144	26.5		399	73.5	
>8	20	42.6		27	57.4	0.003
Household socio-economic status						
Low	26	28.0		67	72.0	
Middle	187	24.5		576	75.5	
High	10	40.0		15	60.0	0.177
Child’s age (months)						
24–59	71	21.3		263	78.7	
12–23	152	27.8		395	72.2	0.031

**Table 6:** Characteristics of children aged 12–59 months with and without stunting in relation to selected socio-demographic and economic characteristics (*n* = 881).

Characteristic	Stunting Status				Bivariate Analysis				Multivariate Analysis			
	Stunted (*n* = 223)		Normal (*n* = 658)		OR	95% CI		*p*	AOR ^†^	95% CI		*p*
	n	%	n	%		Lower	Upper			Lower	Upper	
Marital status of household head												
Married	215	24.9	649	75.1	1.00				1.00			
Otherwise	8	47.1	9	52.9	2.683	1.022	7.041	0.072	1.710	0.628	4.655	0.294
Household size												
<5	59	20.3	232	79.7	1.00				1.00			
5–8	144	26.5	399	73.5	1.149	1.007	2.001		1.706	0.893	3.259	0.106
>8	20	42.6	27	57.4	2.913	1.528	5.552	0.003	2.682	1.359	5.293	0.004
Child’s age (months)												
24–59	152	27.8	395	72.2	1.00				1.00			
12–23	71	21.3	263	78.7	1.425	1.033	1.967	0.031	1.428	1.033	1.973	0.031
Food insecurity												
Food Secure	90	20.4	352	79.6	1.00				1.00			
Mild Food Insecure	47	27.3	125	72.7	1.471	0.978	2.210		1.733	1.021	3.079	0.042
Moderate Food Insecure	29	24.0	92	76.0	1.233	0.765	1.987		1.609	0.987	2.621	0.056
Severe Food Insecure	57	39.0	89	61.0	2.505	1.670	3.756	0.000	2.356	1.540	3.605	0.000
Dietary diversity												
Minimum met	166	25.9	474	74.1	1.00				1.00			
Minimum not met	57	23.7	184	76.3	0.885	0.626	1.250	0.487	0.962	0.671	1.380	0.833

Significance at *p* value of < 0.05, 0.01, and 0.001; 95% CI: 95% confidence interval; OR: odds ratio; ^†^ adjusted with household SES level, child’s age, child’s gender.

## Data Availability

Due to privacy and ethical concerns, neither the data nor the source of the data can be made available.

## Supplementary Materials

The following supporting information can be downloaded at: https://www.mdpi.com/article/10.3390/nu15030573/s1, Figure S1: Frequency and age of children with stunting; Figure S2: Distribution of food groups consumed by children during 24 h before the data collection.
