# Supplementary material for: Dietary Diversity, Household Food Insecurity and Stunting among Children Aged 12 to 59 Months in N’Djamena—Chad
Source: Nutrients. 2023 Jan 21;15(3):573. doi: 10.3390/nu15030573 (PMC9920356; doi:10.3390/nu15030573)
Supplement: Supplementary file 1 [file nutrients-15-00573-s001.zip › nutrients-2175520-supplementary.pdf]

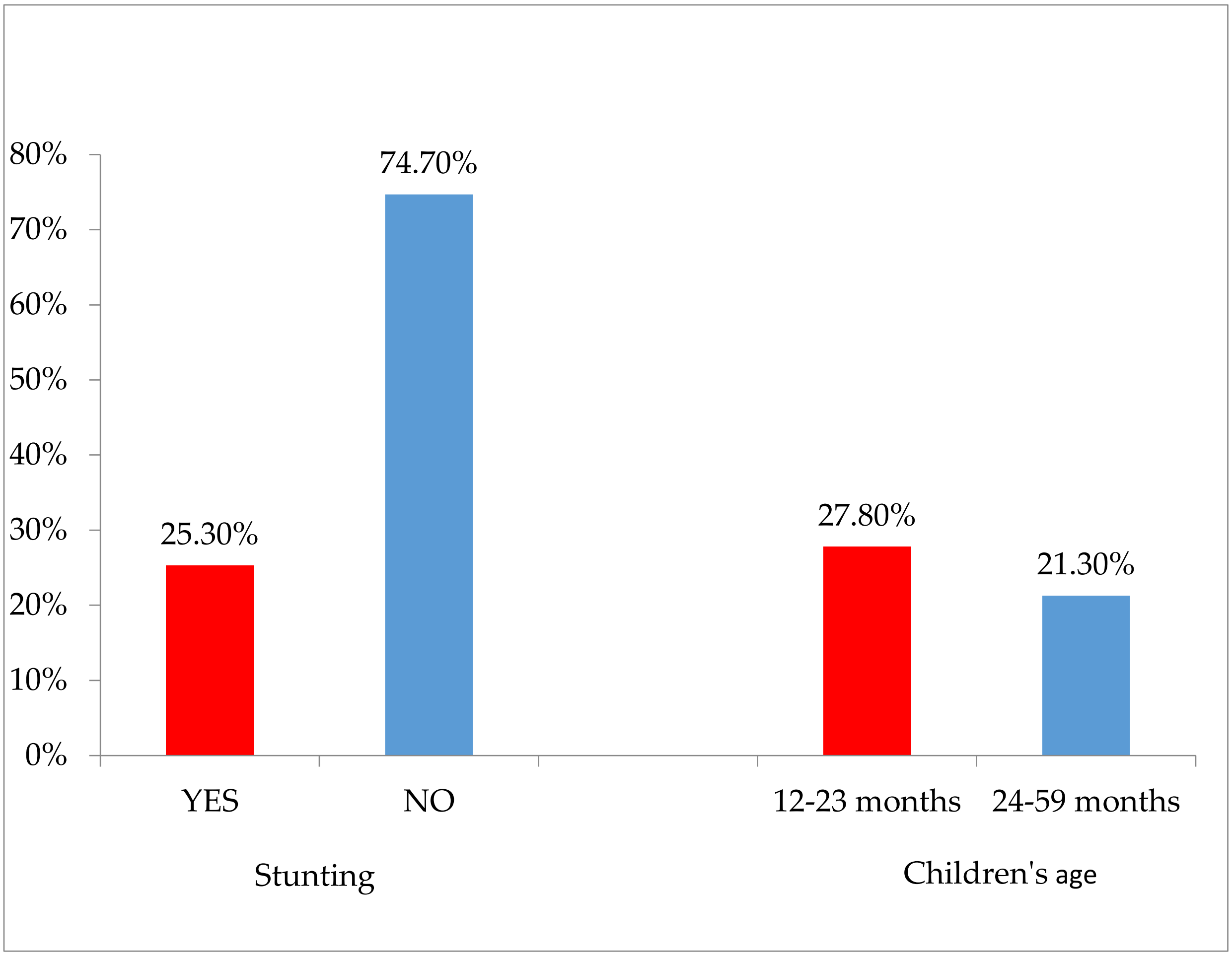

Figure S1. Frequency and age of children with stunting

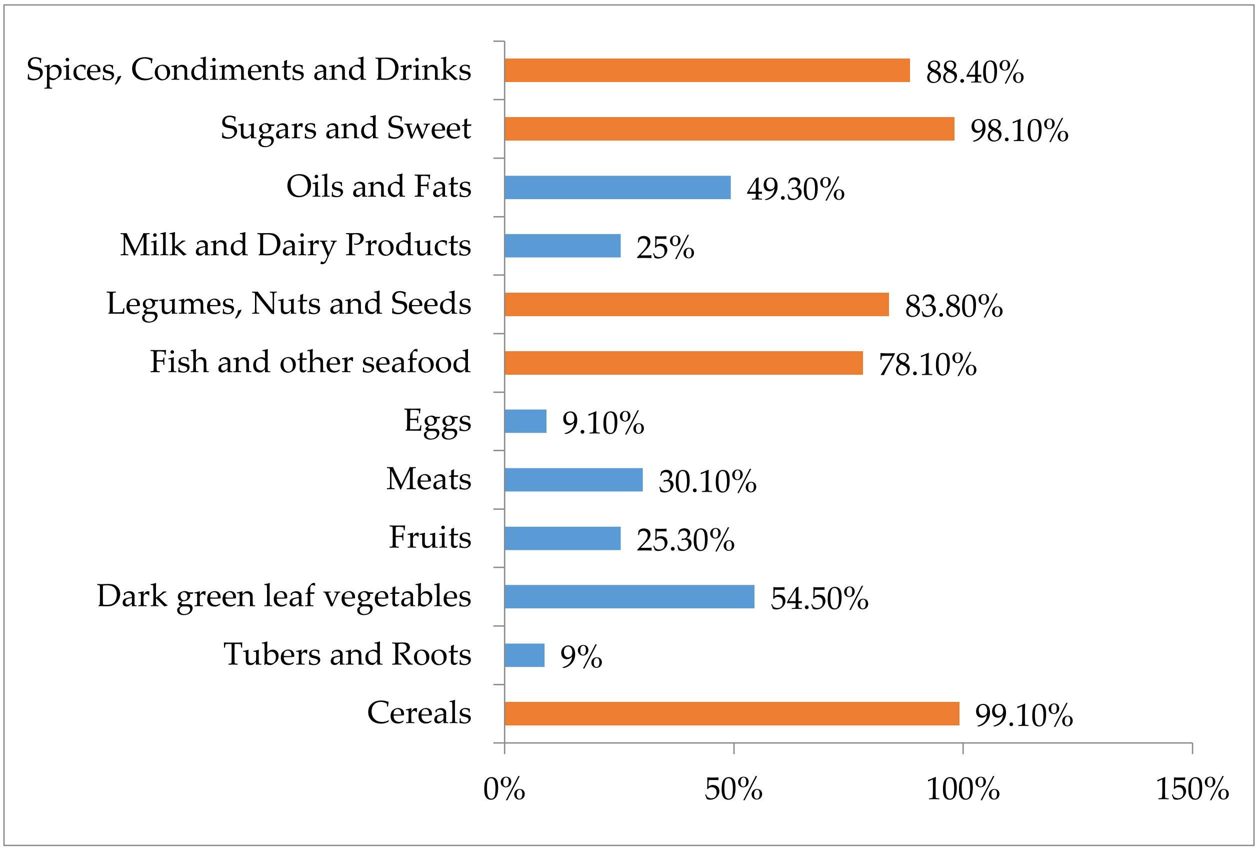

Figure S2. Distribution of food groups consumed by children during 24 h before the data collection
